# Stereotactic Irradiation of GH-Secreting Pituitary Adenomas

**DOI:** 10.1155/2012/482861

**Published:** 2012-02-22

**Authors:** G. Minniti, C. Scaringi, D. Amelio, R. Maurizi Enrici

**Affiliations:** ^1^Department of Neuroscience, Neuromed Institute, 86077 Pozzilli, Italy; ^2^Department of Radiation Oncology, Sant'Andrea Hospital, University Sapienza, 00189 Rome, Italy; ^3^ATreP, Agenzia Provinciale per la Protonterapia, 38122 Trento, Italy

## Abstract

Radiotherapy (RT) is often employed in patients with acromegaly refractory to medical and/or surgical interventions in order to prevent tumour regrowth and normalize elevated GH and IGF-I levels. It achieves tumour control and hormone normalization up to 90% and 70% of patients at 10–15 years. Despite the excellent tumour control, conventional RT is associated with a potential risk of developing late toxicity, especially hypopituitarism, and its role in the management of patients with GH-secreting pituitary adenomas remains a matter of debate. Stereotactic techniques have been developed with the aim to deliver more localized irradiation and minimize the long-term consequences of treatment, while improving its efficacy. Stereotactic irradiation can be given in a single dose as stereotactic radiosurgery (SRS) or in multiple doses as fractionated stereotactic radiotherapy (FSRT). We have reviewed the recent published literature on stereotactic techniques for GH-secreting pituitary tumors with the aim to define the efficacy and potential adverse effects of each of these techniques.

## 1. Introduction

GH-secreting pituitary adenoma is responsible for acromegaly, a disorder characterized by significant morbidity and mortality due to musculoskeletal, cardiovascular, metabolic, and pulmonary complications [1, 2]. 

Surgery, radiotherapy (RT), and medical therapies, including dopamine agonists, somatostatin-receptor ligands, and the GH-receptor antagonist pegvisomant are available treatments for patients with GH-secreting pituitary adenomas. Transsphenoidal surgery is often employed in the initial management of acromegaly. Remission of disease is achieved in up to 60% of patients [3] with a low incidence of surgical complications and significant improvement of metabolic and cardiovascular dysfunctions [4, 5]. Somatostatin analogs are a safe and effective treatment for GH-secreting adenomas, resulting in a normalization of GH/IGF-I hypersecretion in approximately 60–70% of patients [6]. RT is recommended only for patients with persistent active disease after surgery and/or during medical therapy, being able in normalizing GH/IGF-I in up to 70% of patients 10–15 years after RT in several series [7–10]. However, efficacy and potential toxicity of RT remain matter of debate.

More recently, stereotactic radiation techniques have been employed in patients with GH-secreting pituitary adenomas to deliver more localized irradiation with a steeper dose gradient between the tumor and the surrounding normal tissue in order to minimize radiation-induced toxicity while improving its effectiveness [11]. The techniques either involve photon energy from multiple cobalt 60 radiation sources (gamma knife) or a modified linear accelerator (LINAC) and are given as a single fraction stereotactic radiosurgery (SRS) or as fractionated stereotactic radiotherapy (FSRT).

In this paper, we present a critical analysis of the more recent available literature on fractionated stereotactic radiotherapy and radiosurgery for GH-secreting pituitary adenomas, in an attempt to define the efficacy, safety, and role of the individual stereotactic techniques.

## 2. Stereotactic Techniques

### 2.1. Stereotactic Radiosurgery (SRS)

SRS using either a multiple cobalt-60 gamma radiation-emitting sources gamma knife (GK) or a LINAC has been extensively employed in the last two decades in patients with small brain tumors [12]. During SRS, patients are usually immobilized in a fixed frame with a positioning accuracy <1 mm, and a single, high dose of radiation is delivered to a well-defined tumor volume. The GK system is the most widely radiosurgical technique used for treating brain tumors and consists of an array of 201 cobalt-60 sources that are arranged in a hemisphere and focused with a collimator helmet on a single or multiple points termed as isocenters. The best dose conformality is achieved by changing the number and the distribution of isocenters and the beam size by variation of collimator apertures. This results in a sharp-step-dose gradient between the tumor and the surrounding normal tissue, and small target may receive high doses while adjacent radiation-sensitive structures will receive negligible doses.

Instead of using an array of cobalt sources, Linac SRS utilizes X-rays which are derived from colliding accelerated electrons with a target metal. The treatment is delivered with the use of multiple arcs or beams resulting in high-dose differential between the target and normal brain tissue. Isodose gradients are improved by the use of multiple isocentre plans, intensity modulation of the beams, restriction of gantry angles and arc lengths, and microcollimation. Regardless the superiority in terms of dose delivery and distribution claimed for each of these techniques, the reported clinical efficacy and toxicity are similar.

CyberKnife (Accuray, Sunnyvale, CA) is a relatively new technological device which combines a mobile linear accelerator mounted on a robotic arm with an image-guided robotic system. The system allows for frameless SRS achieving the same level of targeting precision as frame-based SRS. Patients are fixed in a thermoplastic mask, and the treatment can be delivered in form of hypofractionated regimen in patients with tumors involving the optic apparatus and who are not suitable for SRS [13, 14].

Particle radiation has been also applied successfully in the treatment of pituitary adenomas. The physical properties of proton irradiation can offer superior conformality in dose distribution when compared to photons, and the advantage becomes more apparent for large volumes [15]. Proton therapy can be delivered as SRS or as FSRT with the same immobilization systems and target accuracy of photon techniques. Although potential benefit that may derives by the irradiation of larger tumors located close to critical anatomic structure, the superiority of protons in terms of clinical efficacy and reduced toxicity remains to be proven.

### 2.2. Fractionated Stereotactic Radiotherapy (FSRT)

FSRT is a stereotactic technique in which a variable number of fractions are delivered to a target by a modified LINAC-based accelerator. Although FSRT uses the same planning system as SRS, patients undergoing FSRT are usually immobilized in a high-precision frameless stereotactic fixation system, including infrared camera guidance [16], dental [17], implanted fiducial markers [18], and mask fixation system [19, 20] with a reported accuracy of 1–3 mm. Thus, FSRT combines the precision of stereotactic technique with the biological advantages of fractionation. Large single doses of radiation are in fact more toxic to normal brain structures than similar doses given in a fractionated manner. FSRT is usually administered in 25–30 daily fractions of 1.8–2 Gy as for conventional RT; however, hypofractionated stereotactic radiotherapy (HSRT) in which a total dose of 20–40 Gy is delivered in 3–7 fractions can be used in selected patients.

The decision on whether to use SRS or FSRT for pituitary tumors mainly depends on the volume of the target lesion and its proximity to sensitive structures. SRS is usually offered to patients with relatively small adenomas not in close proximity of the optic apparatus. A well-defined dose-dependent risk of radiation optic neuropathy exists following single doses of irradiation, and current practice of SRS aims to avoid irradiating the optic apparatus to single doses beyond 10 Gy [21, 22]. Leber et al. [21] reported on 66 patients receiving RS for parasellar tumors. Optic neuropathy occurred in 0%, 26%, and 78% of patients receiving doses less than 10 Gy, 10 to 15 Gy, and more than 15 Gy to the optic apparatus, respectively. Stafford et al. [22] reported an incidence of optic neuropathy of 2% in a series of 215 patients after treatment with SRS for skull base tumors. The rates of optic neuropathy were less than 2% for doses between 8 and 10 Gy and 6.9% for doses in excess of 12 Gy. This means that SRS can be safely employed only for tumors at least 2–4 mm away from the optic chiasm, depending on size, position, and shape of the tumor, as well delivered dose and radiosurgical treatment techniques.

By contrast, there is no restriction to the size of pituitary adenoma suitable for SRT when a conventional fractionation is used, since the delivered total doses are within tolerance of normal brain structures, including the optic apparatus.

## 3. Efficacy and Toxicity of Irradiation

### 3.1. Stereotactic Radiosurgery

Results of 29 recent published studies including 1215 patients with GH-secreting pituitary adenomas treated with SRS are showed in Table 1 [23–51]. At a corrected median followup of 50.6 months (range 6–114 months), the reported tumor growth control following SRS is 98%, ranging from 92 to 100%. A variable biochemical remission of disease ranging from 17% to 82% has been reported, depending by the different lengths of followup and the criteria used to define the biochemical control of disease. When glucose-suppressed plasma GH levels during OGTT and normal age-corrected IGF-I values were used to define the biochemical remission of acromegaly according to recent criteria of endocrinologic cure [52], the 5-year hormonal normalization observed in 9 studies reporting 528 patients was 43% (range 15–60 months) [38, 42, 44–47]. Time to response ranged from 12 to 66 months. GK SRS is the most widely reported radiosurgical technique. Only few studies report on the use of linac SRS for GH-secreting tumors, although with a similar efficacy when compared with GK SRS.

In a retrospective analysis of 83 patients with acromegaly treated with GK SRS at University of Milan San Raffaele between 1994 and 2006, the reported actuarial biochemical remission rates were 30%, 52%, and 85% at 3, 5, and 10 years, respectively [47]. Jezkov$\overset{´}{\text{a}}$ et al. [41] in a series of 96 patients reported hormonal remission rates of 45% at 3 years, 58% at 5 years, and 57% at 8 years, respectively. Similar biochemical remission rates in the range of 45–60% at 5 years have been shown by others [44–46], although lower rates have been reported in some series [36, 38, 42, 51].

Remission of disease after SRS has been associated with pretreatment levels of GH and/or IGF-I levels in some series [38, 41, 44, 47] similar to that reported after conventional RT [7–10]. In a retrospective analysis of 46 consecutive patients 3-year and 5-year biochemical remission rates were 40% and 90% for patients with IGF-I levels less than 2.25 times the upper limit of normal and 23% and 38% with IGF-I levels greater than 2.25 times the upper limit of normal, respectively. Losa et al. [47] reported a median time for remission of 37 months for patients with pretreatment GH levels ≤7 *μ*g/liter as compared with 93 months for patients with GH levels >7 *μ*g/liter. Although no relationship between baseline hormonal levels and remission of acromegaly has been reported in few series [36, 45], it seems reasonable that patients with near-normal GH and IGF-I levels are more likely to achieve hormonal remission than patients with markedly abnormal pretreatment levels.

Marginal doses of 15–34 Gy have been employed for the treatment of GH-secreting pituitary adenomas. In the majority of studies, higher doses were not associated with higher rate of remission or faster normalization of GH/IGF-I levels. Thus, a marginal dose of about 20–25 Gy seems appropriate to achieve either tumor control or hormonal normalization.

The concomitant use of somatostatin analogs at the time of SRS as negative predictor of biochemical remission remains matter of debate. Although in Landolt et al. [25] and Pollock et al. [33], series the use of somatostatin analogs at the time of SRS was correlated with a worse outcome, other authors found no differences in the outcome between patients who received somatostatin agonists at the time of GK and those who did not. Although somatostatin analogs withdrawal before SRS has gained an increase acceptance in clinical practice, future prospective studies are needed to elucidate the issue.

The reported overall rate of serious complications after SRS is low (Table 1). The most commonly observed complication following SRS for secreting adenomas was hypopituitarism, with an incidence ranging between 0 and 69%, although hormonal function has not been systematically evaluated in most studies. In a series of 39 patients with acromegaly treated with GK SRS, a new pituitary deficit occurred in one-third of patients, with an actuarial incidence of hypopituitarism of 10% at 2 years and 33% at 5 years, respectively [27]. Jagannathan et al. [46] reported new endocrine deficiencies in 34% of 95 patients treated with GK SRS, and a similar incidence of hypopituitarism at 5 years has been observed in few other series [41, 45, 48]. Other treatment-related complications occur rarely after SRS. Cranial neuropathies, brain radionecrosis, and carotid artery stenosis have been reported infrequently following SRS. Loeffler et al. [53] reported two cases of secondary brain tumors after SRS for a pituitary adenoma. The risk to develop a new tumor after SRS appears to be significantly less than that seen following fractionated RT [54]; however, the relatively short length of followup of most published series does not allow for any definitive conclusion. 

A recent experience of proton SRS showed a biochemical remission in 50% of 22 patients with GH-secreting pituitary adenomas, with a median time to complete response of 30.5 months [43]. One-third of patients developed at least one new pituitary deficiency, requiring medical therapy. Based on this preliminary experience, the use of proton SRS does not seem to offer clinical advantages when compared to photons SRS.

### 3.2. Fractionated Stereotactic Radiotherapy

Five studies report on the use of FSRT in 115 patients with GH-secreting pituitary adenomas [54–59] (Table 1). At a median corrected followup of 54 months ranging from 28 to 80 months, the reported tumor control was 97%. The median and 5-year biochemical remission of disease were 40% and 42%, being similar to the best reported rates of large studies of conventional RT and SRS. Milker-Zabel et al. [55] reported the normalization of elevated GH level in 70% of 20 acromegalic patients at a median of 26 months, with 5-year local and hormonal control rates of 100% and 80%, respectively. In a series of 18 patients with acromegaly treated with FSRT at Royal Marsden Hospital, biochemical remission was achieved in 35% after a median followup of 39 months [54]. Actuarial normalization of GH/IGF-I levels was 20% at 3 years and 50% at 5 years. At a median followup of 30 months, Roug et al. [59] observed biochemical remission of disease, as defined by suppressed GH at OGTT and normal IGF-I levels adjusted for age, in 30% of 34 patients with active acromegaly, being 24%, 38%, and 64% after 1, 3, and 5 years, respectively.

A low-radiation-induced toxicity has been reported after FSRT, even in the case of large tumors involving the optic apparatus. Hypopituitarism has been reported in 8–37% of patients at median followup ranging from 28 to 82 months, whereas the reported incidence of optic neuropathy is 1–5%. No cases of CVA and second tumors have been reported after FSRT. Since the incidence of such complications increases with time, large series and longer followups need to demonstrate the potential clinical advantages of treating less normal brain at high doses achieved with the use of the stereotactic techniques. Similarly, because of the lack of formal cognitive function testing and quality of life assessment after FSRT, the potential superiority of stereotactic techniques as compared with 3D conformal remains to be clarified.

Initial experiences with the application of CyberKnife in treating patients with acromegaly are promising [60, 61]. In a report of nine patients with acromegaly treated with CyberKnife to doses of 18–24 Gy in one to three fractions, biochemical remission was observed in 4 patients at a mean followup of 25.4 months [61]. Whilst these results are promising, the short followup and the small number of patients do not permit any conclusion about the low risk of optic neuropathy in patients treated with hypofractionated regimens. When fractionation is thought to be safer than SRS, conventional fractionation should be considered on the basis of its proven efficacy and safety.

## 4. FSRS versus SRT

There is much debate about the relative efficacy of SRS and SRT. Currently reported results suggest similar results in terms of tumor control and biochemical remission of acromegaly.

A faster decline of serum GH concentration after GK SRS as compared with FSRT has been reported by some authors [25, 62]. In a small series of 16 patients with acromegaly, Landolt et al. [25] reported mean time to normalization of GH/IGF-I levels of 1.4 years in the group treated with the GK and 7.1 years in the group treated with FSRT. Mitsumori et al. [62] reported mean time to hormone normalization of 8.5 and 18 months in patients treated with SRS and FSRT, respectively. In contrast, recent series have showed a time to hormonal normalization following SRS of 30–66 months [36, 38, 41, 44–47] similar to that reported after fractionated RT [55, 57, 59] and suggesting that time to hormonal normalization is more dependent on preirradiation GH/IGF-I levels than differences in radiation techniques. So far, the superiority of SRS in terms of time to hormonal normalization remains to be demonstrated. Data from stereotactic series suggest that the incidence of hypopituitarism after SRS is lower than that reported after fractionated RT; however, this may reflect different patient selection and length of followups, and large prospective studies are needed to clarify this issue.

In absence of comparative studies, the choice of the radiation technique is based on tumor characteristics. SRS is usually offered to patients with relatively small adenomas less than 3 cm in size and away more than 2-3 mm from the optic chiasm. FSRT should be preferred in patients with large tumors in close proximity of optic apparatus, since the treatment is delivered within the radiation tolerance limits of cranial nerves, including the optic apparatus.

## 5. Conclusion

SRS and FSRT represent effective treatment modalities of irradiation for patients with persistent active GH/IGF-I hypersecretion after surgery and/or during medical therapy, providing a comparable high rates of tumor control and endocrinological remission with low morbidity. Treating less normal brain by higher radiation doses is a clear technical improvement of modern RT which translates into clinical benefit in terms of reduction of late effects of radiation. In most centres, SRS is a convenient approach for patients with relatively small residual GH-secreting tumors, while FSRT is usually reserved to patients with larger tumors not amenable to SRS. Prospective studies comparing SRS with FSRT would be of value to evaluate the long-term efficacy and toxicity of the techniques. Efficacy and toxicity of hypofractionated treatment schedules need to be explored in future studies.

## Figures and Tables

**Table 1:** Summary of results of recent series on SRS for GH-secreting pituitary adenomas.

Authors	patients No	Type of SRS	Total dose Gy	followup median (months)	Tumor control (%)	Hormone normalization (%)	Late toxicity (%)	
							Visual	Hypopituitarism
Morange-Ramos et al. [23]	15	GK SRS	28	20	NA	20	5	16
Lim et al. [24]	20	GK SRS	25	26	92.5	30	5	5
Landolt et al. [25]	16	GK SRS	25	17	NA	50	0	0
Kim et al. [26]	11	GK SRS	28.7	27	NA	35	0	0
Inoue et al. [27]	12	GK SRS	21	>24	94	58	NA	NA
Mokry et al. [28]	10	GK SRS	16	46	100	31	0	NA
Izawa et al. [29]	29	GK SRS	22.5	>6	100	41	0	0
Zhang et al. [30]	68	GK SRS	31	32	NA	40	1.3	4
Landolt et al. [31]	31	GK SRS	25	19.2	NA	69	NA	NA
Ikeda et al. [32]	17	GK SRS	25	58	NA	82	0	0
Pollock et al. [33]	26	GK SRS	20	36	100	47	0	16
Swords et al. [34]	13	LINAC SRS	8–15	25	100	35	0	0
Choi et al. [35]	12	GK SRS	28.5	43	100	30	0	0
Attanasio et al. [36]	30	GK SRS	20	46	100	30 at 5 years	0	6.7
Jane et al. [37]	64	GK SRS	15	>18	NA	36	0	28
Castinetti et al. [38]	82	GK SRS	26	49.5*	NA	17	1.2	17
Gutt et al. [39]	44	GK SRS	23	22	100	48	NA	NA
Kobayashi et al. [40]	67	GK SRS	18,9	63	100	17	11	15
Jezkov$\overset{´}{\text{a}}$ et al. [41]	96	GK SRS	32	53.7	100	44 at 5 years	0	27.1
Voges et al. [42]	64	LINAC SRS	16,5	54.3	97	14 and 33 at 3 and 5 years	1.4	13 and 18 at 3 and 5 years
Petit et al. [43]	22	Proton SRS	20 CGE	75.6	100	59	0	38
Pollock et al. [44]	46	GK SRS	20	63	100	11 and 60 at 2 and 5 years	0	33 at 5 years
Vik-Mo et al. [45]	53	GK SRS	26.5	67	100	58 and 86 at 5 and 10 years	3.8	10 at 5 years
Jagannathan et al. [46]	95	GK SRS	22	57	98	36 and 47 at 3 and 5 years	4	34
Losa et al. [47]	83	GK SRS	21,5	69	97	52 and 85 at 5 and 10 years	0	10 at 10 years
Ronchi et al. [48]	35	GK SRS	20	114	100	15 and 46 at 5 and 10 years	0	69
Wan et al. [49]	103	GK SRS	21,4	67	95	37	0	6
Hayashi et al. [50]	25	GK SRS	25.2	36	100	40	0	0
Iwai et al. [51]	26	GK SRS	20	84	96	17 and 47 at 5 and 10 years	0	8
Milker-Zabel et al. [55]	20	FSRT	52.2	61	100	80 at 5 years	5	15
Colin et al. [56]	31**	FSRT	50.4	80	99	20 and 50 at 5 and 10 years	0	37
Minniti et al.[57]	18**	FSRT	45	39	98	50 at 5 years*	0	22
Imran et al. [58]	12	FSRT	50	28.5	92	33	0	8
Roug et al. [59]	34	FSRT	54	45	91	36 at 5 years	NA	29

NA not assessed.

*mean followup; **acromegalic patients included in series of FSRT for either secreting or non secreting pituitary tumors.
